# The complete chloroplast genome of *Bougainvillea glabra*

**DOI:** 10.1080/23802359.2020.1718028

**Published:** 2020-01-27

**Authors:** Meng He, Xihao Wang, Yangtao Zhuang, Xiang Jin

**Affiliations:** Ministry of Education Key Laboratory for Ecology of Tropical Islands, College of Life Sciences, Hainan Normal University, Haikou, China

**Keywords:** *Bougainvillea glabra*, chloroplast genome, biological diversity, phylogeny

## Abstract

*Bougainvillea glabra* is one of the most popular ornamental and landscaping plants planted in tropical and subtropical regions. The brightly colored bracts, long florescence and strong stress resistance make *B. glabra* perfect ornamental horticulture plant. *Bougainvillea* plants have been frequently hybridized, resulting in more than 400 varieties. To investigate the chloroplast genome will help us to understand the biological diversity and stress resistance of *Bougainvillea* plants better. Here, we report the complete chloroplast genome of *B. glabra,* which is 154,542 bp in length, including a large single copy (LSC) region of 85,695 bp and a small single copy (SSC) region of 18,077 bp, separated by a pair of identical inverted repeat regions (IRs) of 25,385 bp each. A total of 128 genes were identified, including 83 protein-coding genes, 37 tRNA genes, and 8 rRNA genes. Phylogenetic analysis based on 12 chloroplast genomes showed that *B. glabra*, accompanied with its sister species *B. spectabilis,* formed a base clade in Nyctaginaceae which was close to *Pisonia aculeata*. This study will be helpful for better understanding of the genetic diversity and stress resistance of *Bougainvillea* plants.

The genus *Bougainvillea*, which is originated from Amazonian rainforests in South America, contains important ornamental and landscaping plants that are widely distributed in tropical and subtropical areas (Abarca-Vargas and Petricevich 2018). Only four species out of 18 in this genus were well studied: *B. buttiana, B. glabra, B. spectabilis*, and *B. peruviana* (Zhou et al. 2011). In Africa, the extracts of *B. glabra* are used to treat intestinal disorder, inflammation related cases and pain reliever (Ogunwande et al. 2019). In addition, *B. glabra* has gorgeous colored bracts, long florescence and strong stress resistance, which make it high valuable in urban landscape greening and ornamental horticulture (Tsai et al. 2005). Due to its commercialization, the *Bougainvillea* plants were hybridized frequently, resulting in more than 400 varieties, the genetic background of which are complex (Zhao et al. 2019). Chloroplasts are important for plant interactions with the environment (heat, drought, salt, light, etc.) and chloroplast genome sequence is also helpful to understand biological diversity (Daniell et al. 2016). Here, we report the complete chloroplast genome of *B. glabra,* which will be useful to understand the biological diversity and stress resistance of *Bougainvillea* species, accompanied with recently reported *B. spectabilis* chloroplast genome (Wang et al. 2019; Yao et al. 2019).

The sample of *B. glabra* was collected from the botanical garden of Hainan Normal University, Haikou, China (100°20′35.63″E, 19°59′46.72″N). The voucher specimen is stored in the herbarium of Hainan Normal University (specimen no. 19HNNU0722). Chloroplasts were isolated from 30 g of fresh leaves by gradient centrifugation on Percoll. Then, chloroplast genomic DNA were extracted using SDS method. A 350 bp DNA library was constructed and then sequenced PE-150 bp on Illumina NovaSeq 6000 platform. A total of 2.3 GB clean data were generated. Complete chloroplast genome of *B. glabra* was assembled by SOAPdenovo 2.04. Assembled chloroplast genome was annotated using online tool CPGAVAS (http://www.herbalgenomics.org/cpgavas/) (Shi et al. 2019).

The complete chloroplast genome of *B. glabra* (GenBank Accession MN888961) has a circular DNA of 154,542 bp with GC content of 35.19%. It has a large single copy (LSC) region of 85,695 bp and a small single copy (SSC) region of 18,077 bp, separated by a pair of identical inverted repeat regions (IRs) of 25,385 bp each. In addition, we annotated 128 genes, including 83 protein-coding genes, 37 tRNA genes, and 8 rRNA genes.

To reveal the phylogenetic position of *B. glabra*, a maximum-likelihood (ML) phylogenetic tree was constructed using 12 complete chloroplast genome sequences, including *B. glabra*, 9 *Caryophyllales* species and two *Buxaceae* outgroups (*Buxus microphylla* and *Pachysandra terminalis*). All sequences were download from GenBank under corresponding accessions and were aligned using MAFFT v7.455 (Katoh and Standley 2013). The ML tree was produced by RAxML (Stamatakis 2014) using 1000 bootstrap replicates. The phylogenetic tree showed that *B. glabra*, accompanied with *B. spectabilis*, formed a base clade in Nyctaginaceae which was close to *Pisonia aculeata* (Figure 1). The complete chloroplast genome of *B. glabra* will help to study the genetic diversity and stress resistance of *Bougainvillea* plants.

**Figure 1.**
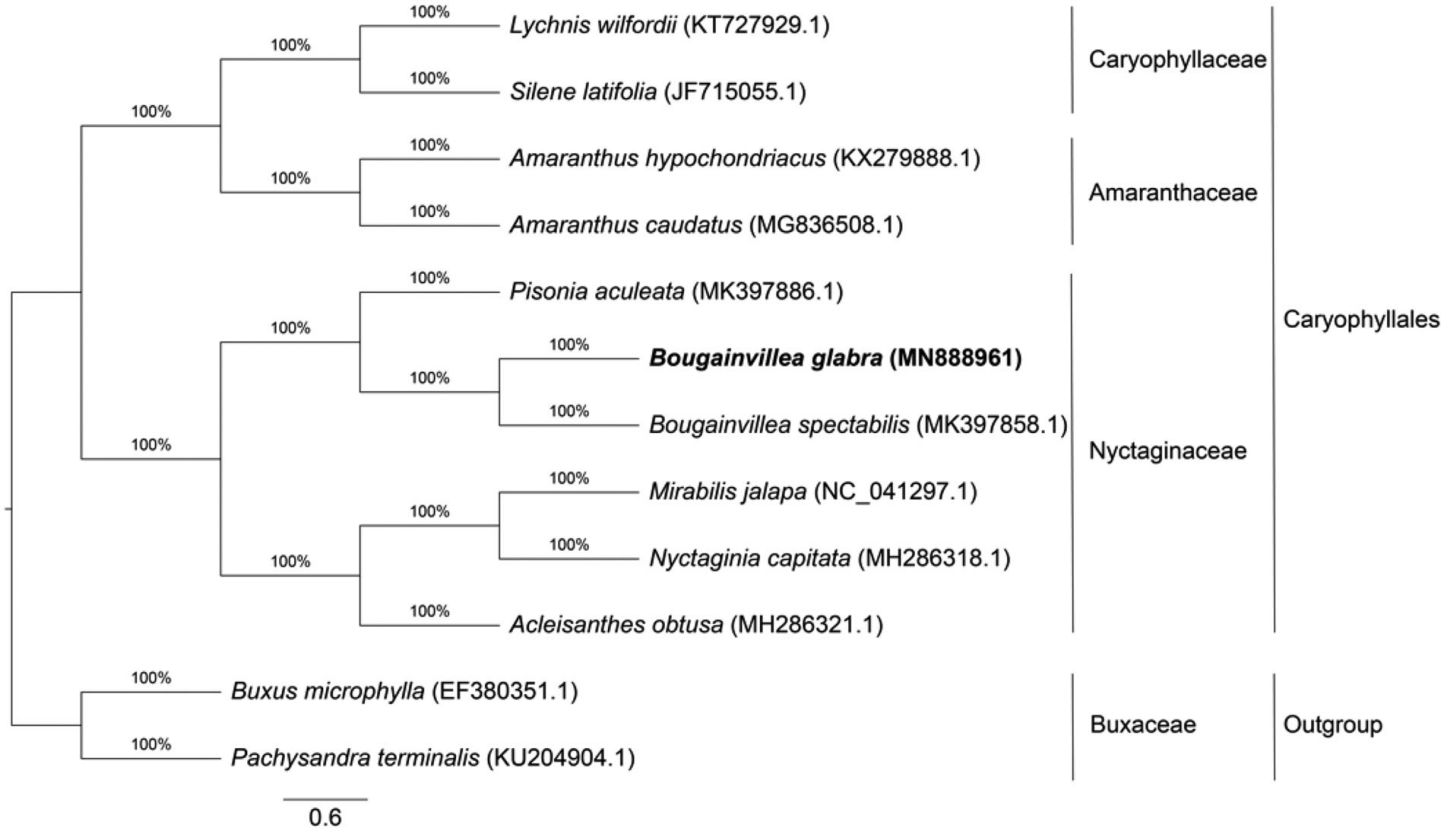
Phylogenetic analysis of 10 species from *Caryophyllales* and two outgroups based on complete chloroplast genome sequences by RAxML. Bootstrap was set to 1000.
